# Genomic Selection in Rice Using Targeted Sequencing: Model Evaluation and Training Population Optimization for Breeding Applications

**DOI:** 10.3390/genes17101240

**Published:** 2026-10-08

**Authors:** Zaijie Chen, Leibo Pan, Yue Wang, Min Lin, Yan Lin, Mingji Wu, Huaqing Liu, Taijiao Hu, Shaohua Yang, Yana Song

**Affiliations:** Fujian Key Laboratory of Agricultural Genetic Engineering, Institute of Biotechnology, Fujian Academy of Agricultural Sciences, Fuzhou 350003, China; czj@cropdesign.cn (Z.C.); panleibo_7481@163.com (L.P.); wy@cropdesign.cn (Y.W.); linmin_0916@163.com (M.L.); ly@cropdesign.cn (Y.L.); wmj@cropdesign.cn (M.W.); lhq@cropdesign.cn (H.L.); htj@cropdesign.cn (T.H.)

**Keywords:** rice, genomic selection, targeted sequencing, training population optimization, genome-wide association study, genomic prediction

## Abstract

Background/Objectives: Genomic selection (GS) can accelerate crop improvement, but its application in breeding programs is often constrained by the cost of high-density genotyping. This study evaluated genomic prediction using 4647 targeted sequencing-derived SNPs in 829 rice accessions and investigated the effects of prediction model selection, population structure, training population optimization, and GWAS-assisted marker selection on predictive performance. Methods: Seventeen GS models were evaluated for eight agronomic traits using repeated five-fold cross-validation, cross-population prediction, STPGA-based training population optimization, and GWAS-derived marker subsets. Resaults: Predictive performance varied substantially among traits and models. For grain length (GL), EnsembleGS achieved the highest predictive performance among the 17 models (*r* = 0.838), while RKHS showed the highest performance for grain number per panicle (*r* = 0.698), RFR for heading date (*r* = 0.854), and EnsembleGS for length-to-width ratio (*r* = 0.844). Cross-population prediction generally resulted in lower predictive performance than within-population cross-validation, with substantial variation among transfer directions. STPGA retained substantial predictive performance with reduced training population sizes; for GL, the highest performance reached *r* = 0.800 with a 400-individual STPGA-selected training population. GWAS-guided marker prioritization also retained substantial predictive information after marker reduction. For GL, the highest performance reached *r* = 0.789 and 0.802 using the GWAS-Top500 and GWAS-Top1000 subsets, respectively, compared with 0.751 and 0.775 for the corresponding Random500 and Random1000 subsets. For length-to-width ratio, the corresponding values were *r* = 0.764 and 0.800 for GWAS-Top500 and GWAS-Top1000, compared with 0.727 and 0.783 for the random subsets. Conclusions: Integrating targeted sequencing with trait- and model-aware prediction, optimized training population design, and GWAS-guided marker prioritization can retain substantial genomic prediction performance while reducing marker and training population requirements, providing a potentially cost-effective framework for genomic selection in rice breeding.

## 1. Introduction

Genomic selection (GS) is a genomic prediction approach that uses genome-wide marker information to estimate breeding values, and has become a core technology in modern molecular breeding since its introduction by Meuwissen et al. in 2001 [1]. By integrating genome-wide marker data, GS enables the early prediction of genetic potential in breeding materials, thereby improving selection efficiency and significantly shortening breeding cycles [2]. With the development of high-throughput genotyping technologies, GS allows the early-generation selection of promising individuals, reducing reliance on large-scale field phenotyping. However, the predictive performance of GS models is influenced by multiple factors, including marker density, trait genetic architecture, population structure, and the composition of the training population [3].

Compared to whole-genome resequencing, targeted sequencing provides a cost-effective, high-throughput, and breeding-friendly genotyping strategy, making it increasingly attractive for large-scale breeding programs [4]. However, because targeted sequencing typically generates a limited number of SNP markers, its effectiveness for genomic prediction remains to be fully evaluated. In practical breeding applications, cost constraints make high-density genotyping difficult to implement at scale; therefore, low-density marker strategies are widely adopted [3,5]. In this context, the trade-off between marker density and prediction accuracy becomes a key issue in GS applications. For traits with high heritability or controlled by a limited number of major-effect loci, low-density markers may still capture most of the genetic variation and achieve satisfactory prediction accuracy. In contrast, for complex quantitative traits controlled by numerous small-effect loci, reduced marker density may not adequately capture genome-wide genetic variation, thereby limiting predictive performance [6,7]. Therefore, systematic evaluation of GS performance under low-density SNP conditions is essential for guiding practical breeding strategies.

Beyond marker density, population structure is another critical factor affecting GS accuracy. Genetic stratification can reduce the relatedness between training and testing populations, often leading to substantially decreased predictive ability in cross-population scenarios [8,9]. To address this issue, optimizing the composition of the training population to better represent the genetic diversity of the target population has been proposed as an effective strategy to improve prediction accuracy. In particular, training population optimization methods based on algorithms such as STPGA have shown strong potential in improving the representativeness of training sets [10,11].

Genome-wide association studies (GWASs) provide an important tool for dissecting the genetic basis of complex traits. By identifying loci significantly associated with trait variation and incorporating these informative markers into GS models, it is possible to improve the capture of key genetic signals under limited marker density, thereby mitigating information loss and enhancing prediction efficiency [12,13,14]. However, few studies have systematically evaluated GS performance using low-density targeted sequencing data while simultaneously integrating training population optimization and GWAS-assisted marker selection, and their combined effects remain unclear. More importantly, existing studies have generally evaluated these strategies independently, whereas their potential synergistic effects under breeding-oriented targeted sequencing platforms have rarely been investigated. Therefore, a comprehensive evaluation framework integrating marker system, training population optimization, and GWAS-assisted marker prioritization is needed to support cost-effective genomic selection in practical breeding programs.

Therefore, in this study, we used targeted sequencing data from 829 rice accessions to systematically evaluate the predictive performance of 17 GS models under low-density SNP conditions. We further investigated the effects of population structure, STPGA-based training population optimization, and GWAS-assisted marker selection on genomic prediction accuracy. Unlike previous studies focusing on individual optimization strategies, this study provides an integrated evaluation of breeding-oriented genomic selection by combining a targeted sequencing platform, optimized training population design, and trait-informed marker prioritization within a unified analytical framework. This study aims to develop a cost-effective genomic selection strategy for practical breeding applications, providing theoretical and methodological support for efficient molecular breeding in rice.

## 2. Materials and Methods

### 2.1. Plant Materials and Phenotypic Data

A total of 829 rice accessions were used in this study, including 210 elite cultivars from southern China and 619 breeding intermediate lines. All accessions were evaluated under field conditions in Fuzhou, Fujian Province, China, during the 2022 growing season. The materials were cultivated under a uniform field management regime. Each accession was planted in an individual plot consisting of five rows with eight plants per row, resulting in 40 plants per accession, with approximately 15 cm spacing between adjacent plants. No independent field replicates or formal block design were established, and the accessions were not randomly arranged across the field. Therefore, the experiment represented a single-environment field evaluation rather than a replicated randomized field trial.

Phenotypic data were collected for eight agronomic traits, including heading date (HD, days), grain number per panicle (GN, grains panicle^−1^), seed setting rate (SR, proportion), grain length (GL, mm), grain width (GW, mm), length-to-width ratio (LWR, dimensionless), thousand-grain weight (TGW, g), and panicle number (PN, panicles plant^−1^). For each accession, the five plants located in the central portion of the plot were selected for phenotypic evaluation. All panicles from these five plants were collected for subsequent grain-related measurements to reduce the potential influence of plot-border effects.

Panicle number (PN) was manually counted for the five sampled plants. The total grain number and filled grain number from all collected panicles were measured using an automatic rice grain analysis system. Seed setting rate (SR) was calculated as the proportion of filled grains among the total grains. Grain number per panicle (GN) was calculated as the total number of filled grains divided by the corresponding panicle number. Grain length (GL), grain width (GW), length-to-width ratio (LWR), and thousand-grain weight (TGW) were obtained from the automatic rice grain analysis system. Heading date (HD) was recorded during field observation according to the heading status of each accession. Measurements from the sampled plants and their panicles were summarized to obtain a single accession-level phenotypic value for each trait.

The accession-level phenotypic values were directly used as response variables in the genomic selection analyses. Specifically, the GS models were trained using the observed accession-level phenotypic values rather than BLUEs, BLUPs, breeding values, or other variance-component-adjusted phenotypes. Because the phenotypic evaluation was conducted at a single location and in a single growing season without independent field replication, the experimental design did not provide sufficient replication to reliably separate genetic and environmental variance components or to obtain adjusted BLUE or BLUP values. Therefore, no additional mixed-model adjustment or BLUP estimation was applied.

Missing phenotypic observations were handled using a k-nearest neighbors (KNN) imputation procedure before downstream analyses. KNN imputation was performed separately for each trait using the available phenotypic information, and the resulting dataset contained phenotypic values for all 829 accessions. The KNN-imputed dataset was used consistently for the descriptive statistics and for the subsequent genomic selection analyses. The number and proportion of observations affected by KNN imputation for each trait are summarized in the descriptive-statistics table.

Because the experiment was conducted in a single environment without independent field replication, conventional heritability estimation based on a replicated experimental design was not performed. The observed and KNN-imputed phenotypic values therefore represent accession performance under the specific environmental conditions of Fuzhou in 2022 and may contain both genetic and environmental components of variation. The use of KNN imputation was intended to address missing observations and does not constitute an adjustment for environmental or experimental effects.

The single-location, single-season, and non-replicated design also limits the separation of genetic effects from environmental and residual variation and does not allow genotype × environment (G × E) interactions to be evaluated. Because no independent replicated plots or formal block structure were established, block effects could not be reliably estimated. Likewise, the available experimental design did not provide sufficient spatial replication or a suitable field layout for robust spatial-effect adjustment. Accordingly, no additional block- or spatial-effect mixed model was fitted. The genomic prediction results should therefore be interpreted as predictive performance under the specific phenotypic evaluation conditions of Fuzhou in 2022, rather than as direct estimates of breeding-value prediction across multiple environments or years.

### 2.2. Genotypic Data

A targeted sequencing panel was first developed to capture genome-wide background polymorphisms together with functionally relevant and previously reported trait-associated variants. The panel was constructed based on polymorphic sites derived from the 3K Rice Genomes Project and MNP markers, as well as reported functional variants, GWAS-associated loci, and transgenic/gene-editing sites. During panel design, candidate variants were screened based on minor allele frequency (MAF ≥ 0.05), missing rate (≤10%), and sequencing depth (≥5×), followed by linkage disequilibrium (LD) pruning (r^2^ < 0.8). A sliding-window strategy of approximately 200 bp was used to integrate neighboring polymorphic sites. Candidate regions containing multiple high-quality markers and exhibiting high polymorphism information content were preferentially retained. Functional and GWAS-associated variants were selected from previously reported genes and loci, with redundant markers in high-LD regions removed.

After construction of the targeted sequencing panel, genomic DNA from the 829 rice accessions was used for targeted sequencing. Following DNA quality assessment, sequencing libraries with an insert size of approximately 300–500 bp were prepared using an enzymatic fragmentation-based method. Libraries were sequenced on the BGI/MGI BGISEQ-2000RS platform using paired-end 150 bp sequencing (PE150). The resulting sequencing data were generated in FASTQ format (.fq.gz).

Raw sequencing reads were initially evaluated using FastQC v0.11.9 to assess base quality, GC content, sequence duplication, and potential contamination. Adapter sequences, low-quality bases, and contaminating sequences were subsequently removed using the BBDuk module of BBMap v39.01. The resulting high-quality reads were aligned to the Nipponbare T2T-NIP/AGIS-1.0 reference genome using BWA v0.7.17. SAMtools v1.19 was used for alignment file processing, including SAM-to-BAM conversion and sorting, generating standardized BAM files for downstream variant calling.

Variant detection was performed using the HaplotypeCaller module of GATK v4.5.0.0. Individual-sample variant information was first generated in GVCF format, followed by joint genotyping across samples to obtain a multi-sample VCF file. For the genotype dataset obtained from the 829 accessions, SNPs were subsequently filtered using VCFtools v0.1.17. Variants with MAF < 0.05 or a missing genotype rate > 20% were removed, retaining SNPs with MAF ≥ 0.05 and a missing genotype rate ≤ 20%. This final filtering threshold was applied to the called genotype dataset and was independent of the ≤10% missing-rate criterion used during the earlier targeted-panel design stage. Genotype imputation was subsequently performed using Beagle v5.2 to infer missing genotypes and improve genotype completeness. The resulting high-quality SNP dataset was used for downstream population structure analysis, genomic prediction, GWAS, and training-population optimization analyses.

### 2.3. Genomic Selection Models

Seventeen genomic selection (GS) models were evaluated, representing six model categories: linear models, Bayesian models, kernel-based models, machine-learning models, deep-learning models, and ensemble models (Table 1). The analyses were conducted using the MultiGS platform [15], with model-specific implementations and parameter settings summarized in Supplementary Table S7.

The linear and Bayesian models were implemented using the R-based MultiGS-R framework. RR-BLUP was fitted using a mixed-model framework, while GBLUP used a genomic relationship matrix constructed according to the VanRaden method. BRR, BayesA, BayesB, BayesC, and Bayesian Lasso were implemented using their corresponding Bayesian formulations. RKHS was used as the kernel-based genomic prediction model. The machine-learning and deep-learning models were implemented using the corresponding components of the MultiGS platform. The machine-learning models included random forest regression (RFR), support vector regression (SVR), XGBoost, and LightGBM, while the deep-learning models included DNNGS, MLPGS, DeepResBLUP, and DeepBLUP. The specific software implementations, model architectures, priors, regularization settings, optimization parameters, and training settings are provided in Supplementary Table S7.

Model parameters were specified before model evaluation and were kept fixed and consistent across validation folds for each model. The predefined parameter settings were applied uniformly across the repeated cross-validation analyses, rather than performing extensive model-specific or trait-specific hyperparameter optimization. Thus, the comparison was conducted under a standardized parameter-setting framework, with the detailed model-specific parameter and training settings provided in Supplementary Table S7. No information from the validation data was used to determine model parameters or fit the corresponding prediction models. Thus, model evaluation was performed independently within each training–validation split, avoiding the use of validation data for model fitting.

EnsembleGS combined predictions from five component models, including RR-BLUP, BRR, LightGBM, MLPGS, and DeepBLUP. Within each cross-validation fold, the component models were fitted using only the training data and generated predictions for the corresponding validation data. The ensemble prediction was then obtained by combining the component-model predictions according to the predefined ensemble procedure. No independent inner validation set was used to estimate ensemble weights, and the validation observations used for evaluating the ensemble were not used to fit the component models or estimate the ensemble parameters. The detailed ensemble construction and parameter settings are provided in Supplementary Table S7.

For all models, predictive performance was evaluated using Pearson’s correlation coefficient (*r*) between observed and predicted phenotypic values. A five-fold cross-validation scheme was repeated independently three times using the same training–validation partitions across models to facilitate a consistent comparison of predictive performance.

### 2.4. Cross-Validation Strategy

In the initial model evaluation, five-fold cross-validation was performed to assess the predictive performance of all genomic selection (GS) models using the full population of 829 rice accessions. The five-fold cross-validation procedure was independently repeated three times using different random seeds to generate independent data partitions, resulting in 15 train–test partitions for each trait × model combination. Predictive performance was quantified using the Pearson correlation coefficient (*r*) between observed and predicted phenotypic values for each train–test partition. The resulting mean and standard deviation (SD) were calculated across the 15 cross-validation results to describe average predictive performance and variation among the train–test partitions.

For subsequent cross-population prediction analyses, predictive performance was evaluated using the Pearson correlation coefficient (*r*) between observed and predicted phenotypic values. The predefined genetic subpopulations identified by K-means clustering were used directly as training and testing populations according to the cross-population prediction schemes described in Section 2.6.

For training population optimization, STPGA-selected training populations were generated independently using 10 different random seeds for each predefined training population size (100, 200, 300, 400, and 500 individuals). Each independent STPGA run therefore generated a separate training population of the specified size. Predictive performance was calculated separately for each STPGA run using the Pearson correlation coefficient (*r*) between observed and predicted phenotypic values. The results were summarized across the 10 independent STPGA runs using the mean and standard deviation (SD) to characterize the average predictive performance and between-run variation.

For comparison, randomly selected training populations were independently generated 100 times for each training population size. The same prediction framework and predictive performance metric were applied to each random replicate, and predictive performance was summarized using the mean and SD across the 100 repetitions. Thus, both STPGA and random sampling were evaluated using repeated independent sampling, while the larger number of random repetitions was retained to characterize the distribution of predictive performance under random sampling.

### 2.5. Population Structure Analysis

Principal component analysis (PCA) was performed to characterize the genetic structure of the 829 rice accessions based on the genome-wide SNP dataset. PCA was conducted using PLINK v1.9, and the first three principal components (PC1, PC2, and PC3) were used to visualize the major patterns of genetic variation among the accessions.

To define genetic subgroups for subsequent cross-population prediction analyses, K-means clustering was performed using the PCA scores. The optimal number of clusters was evaluated using the elbow criterion based on the within-cluster sum of squares (WCSSs). K = 3 was selected because the reduction in WCSS became less pronounced beyond three clusters. The resulting three clusters were designated as subgroups A, B, and C and contained 314, 317, and 198 individuals, respectively.

Importantly, the K = 3 classification was used as an operational grouping for the subsequent cross-population prediction analysis and was not intended to imply the existence of exactly three discrete biological populations. The observed genetic structure was interpreted as a simplified representation of the continuous genetic variation captured by the SNP dataset.

### 2.6. Phylogenetic Tree Construction

A phylogenetic tree was constructed to characterize the genetic relationships among the 829 rice accessions using the same set of 4647 high-quality SNP markers used for the population structure analysis. The SNP genotype matrix was converted to PHYLIP format, resulting in an alignment containing 829 sequences and 4647 nucleotide sites. Maximum-likelihood (ML) phylogenetic inference was performed using IQ-TREE v3.0.1. The best-fit nucleotide substitution model was selected using ModelFinder based on the Bayesian information criterion (BIC), and GTR+F+I+R8 was selected as the best-fit model. Branch support was assessed using 1000 ultrafast bootstrap (UFBoot) replicates and 1000 SH-aLRT replicates. The resulting phylogenetic tree was exported in Newick format and subsequently visualized and annotated using the Interactive Tree of Life (iTOL) web server.

The phylogenetic tree was used as an independent representation of genetic relatedness among the accessions and to examine the consistency between phylogenetic relationships and the population structure identified by PCA and K-means clustering. The K-means-defined subgroups were not determined from the phylogenetic tree. Instead, PCA followed by K-means clustering was used to define the operational genetic subgroups for subsequent cross-population prediction analyses, whereas the phylogenetic tree was used to provide an independent visualization of genetic relationships among the accessions. Therefore, the phylogenetic tree was not interpreted as evidence for the existence of three strictly discrete biological populations.

### 2.7. Training Population Optimization Using STPGA

To optimize training populations for genomic selection, the stochastic selection method based on genetic algorithm (STPGA) was applied [10]. STPGA uses a genetic algorithm to identify training populations that minimize the expected prediction error variance (PEV) for the corresponding target population, thereby reducing uncertainty in genomic prediction. The first 50 principal components (PCs) derived from the genome-wide marker matrix were used to characterize the genetic structure of the 829 rice accessions during training population optimization. Five predefined training population sizes (100, 200, 300, 400, and 500 individuals) were evaluated.

The complete set of 829 rice accessions was defined as the candidate population for STPGA optimization. For each predefined training population size, STPGA independently selected the corresponding number of accessions from the 829 candidate individuals to form the training population. The remaining unselected accessions were defined as the prediction target for that specific training population size. Thus, both the training population and its corresponding target population varied with training population size. The predictive performance at each training population size was therefore evaluated for the corresponding STPGA-selected training–target configuration rather than as a strictly controlled comparison using an identical target population.

STPGA optimization was performed using PEV as the primary optimization criterion. A ridge-regression regularization parameter of 1 × 10^−9^ was used. For each training population size, the genetic algorithm was independently run 10 times using different random seeds to account for the stochastic nature of the optimization procedure. The genetic algorithm settings and other STPGA parameters used in this study are summarized in Supplementary Table S8.

The selected training populations from the independent STPGA runs were subsequently evaluated using genomic prediction, with the corresponding remaining accessions serving as the prediction targets. Predictive performance across the independent runs was summarized to characterize the performance and variability of the STPGA-selected training populations.

#### 2.7.1. Prediction Error Variance (PEV)

PEV was used as the primary optimization criterion for STPGA training population selection and was calculated according to the STPGA framework described by Akdemir et al. [10]. Lower PEV values indicate lower expected prediction uncertainty and, consequently, greater expected predictive efficiency. PEV was calculated as

$$PEV(S)=\sigma_{g}^{2}\left(K_{SS}-K_{ST}K_{TT}^{-1}K_{TS}\right)$$

where $K_{TT}$, $K_{SS}$, and $K_{ST}$ denote the genomic relationship submatrices for the training population, testing population, and their cross-relationships, respectively, and $\sigma_{g}^{2}$ represents the additive genetic variance.

For each predefined training population size, STPGA was independently run 10 times using different random seeds. The resulting PEV values were used to characterize the optimization performance and between-run variation in the selected training populations.

#### 2.7.2. PCA-Based Characterization of Selected Training Populations

Following STPGA selection, the genetic representation of the selected training populations was further characterized in the principal component (PC) space derived from the complete 829-accession population. PCA was performed using the 4647 high-quality SNP markers, and the same PC space was used for all STPGA runs and training population sizes.

PC coverage was used as an auxiliary descriptive measure rather than as an optimization criterion. It was calculated as the proportion of the variance represented by the selected training population relative to that of the complete population across the first *n* principal components:

$${Coverage}_{PC}=\frac{\sum_{i=1}^{k}Var(X_{PC}[T,i])}{\sum_{i=1}^{k}Var(X_{PC}[:,i])}$$

where $X_{PC}[T,i]\;$ denotes the scores of the selected training individuals on the *i*-th principal component, $X_{PC}[:,i]$ represents the corresponding variance of the complete population, and *n* represents the number of principal components included in the calculation.

PC coverage was used only to describe the genetic representativeness of the selected training populations and was not used to determine the STPGA optimization objective or to directly calculate genomic prediction performance. Detailed STPGA settings, including the candidate population, training population sizes, number of independent runs, number of PCs, genetic algorithm parameters, optimization criterion, regularization parameter, and prediction-target definition, are provided in Supplementary Table S8.

### 2.8. Genome-Wide Association Analysis

Genome-wide association analysis (GWAS) was performed to identify SNPs associated with the eight target traits using the Bayesian-information and Linkage-disequilibrium Iteratively Nested Keyway (BLINK) method [25]. The GWAS was conducted using the 400 accessions selected by STPGA as the training population for the subsequent marker-prioritization analysis. Phenotypic values for each trait and the corresponding filtered SNP genotype matrix were used as inputs. Manhattan plots were generated using a nominal threshold of *p* < 10^−5^ to highlight relatively strong or suggestive association signals. Because the objective of the GWAS was to prioritize potentially informative markers for genomic prediction rather than to perform formal hypothesis testing, SNPs were subsequently ranked according to their association *p*-values without applying a multiple-testing correction.

### 2.9. GWAS-Based Multi-Trait SNP Prioritization

To identify informative markers for genomic prediction under low-density SNP conditions, GWAS results from all eight target traits were integrated using a multi-trait weighted scoring strategy. Trait-priority weights were predefined before SNP ranking based on practical rice breeding experience and the breeding objectives considered in this study (Table 2). The weights were intended to represent the relative priority of individual traits for breeding-oriented marker selection, rather than quantitative effect sizes or statistically estimated parameters. Accordingly, GN, TGW, and PN were assigned the highest priority (weight = 2.0), SR was assigned an intermediate–high priority (weight = 1.5), GL and GW were assigned an intermediate priority (weight = 1.0), and LWR and HD were assigned a lower priority (weight = 0.5). These weights were used solely for multi-trait SNP prioritization and did not represent phenotypic measurement weights or weights estimated from the GWAS results.

For each SNP and trait combination, the GWAS *p*-value was first transformed using $-{log}_{10}P$ to represent the strength of the association signal. The resulting values were then standardized separately for each trait to make association signals comparable across traits. Specifically, the standardized score for SNP (*i*) and trait (*j*) was calculated as

$${\mathrm{Score}}_{i}=\sum_{j=1}^{8}w_{j}\left(\frac{-{log}_{10}(P_{ij})-\mu_{j}}{\sigma_{j}}\right)$$

where $P_{ij}$ denotes the GWAS *p*-value of SNP *i* for trait *j*; $\mu_{j}$ and $\sigma_{j}$ represent the mean and standard deviation of the $-{log}_{10}(P)$ values for trait *j*, respectively; and $w_{j}$ denotes the weight assigned to trait *j*. Standardization was performed to make the GWAS signals across different traits comparable before their integration according to the predefined trait priorities.

SNPs were ranked in descending order according to their integrated scores. SNPs with higher scores were considered to have greater overall priority across the eight target traits under the predefined breeding objectives. The top 500 and top 1000 ranked SNPs were selected to construct reduced marker panels for subsequent genomic prediction analyses. The numerical values of the weights were used to encode relative priority levels (high, intermediate–high, intermediate, and low), rather than to indicate proportional differences in trait effects or breeding value contributions.

### 2.10. Random SNP Subset Selection as a Marker-Reduction Control

To determine whether the genomic prediction performance of the GWAS-prioritized SNP subsets resulted from trait-informed marker prioritization rather than simply from reducing the number of markers, random SNP subsets were generated as controls. For each marker density, 500 and 1000 SNPs were randomly sampled from the complete set of 4647 SNPs, corresponding to the Random500 and Random1000 subsets, respectively. Random sampling was independently repeated 100 times for each marker density to account for variation among randomly selected marker subsets.

For each random replicate, genomic prediction was performed using the same prediction framework and cross-validation procedure as that used for the corresponding GWAS-prioritized SNP datasets. Predictive performance was quantified using Pearson’s correlation coefficient (*r*) between observed and predicted phenotypic values. The distributions of predictive performance across the 100 random subsets were summarized and compared with those obtained using the GWAS-prioritized Top500 and Top1000 SNP subsets.

The complete 4647-SNP dataset was retained as the full-marker baseline. Thus, five marker-density conditions were evaluated: Full4647, Random1000, GWAS-Top1000, Random500, and GWAS-Top500. The 400 accessions selected by STPGA were used as the training population for the GWAS-based marker-prioritization analysis, with the corresponding remaining 429 accessions serving as the prediction target. This design allowed the performance of GWAS-prioritized marker subsets to be evaluated relative to both the full marker set and random marker-reduction controls while maintaining the same training–target population configuration.

## 3. Results

### 3.1. Phenotypic Variation in the Eight Agronomic Traits

The eight agronomic traits exhibited substantial phenotypic variation across the 829 rice accessions (Table 3). The coefficients of variation (CV) ranged from 9.05% for grain length (GL) to 26.76% for grain number per panicle (GN), indicating considerable phenotypic diversity within the population. Yield-related traits, including grain number per panicle (GN) and panicle number (PN), exhibited relatively greater phenotypic variation (CV = 26.76% and 26.57%, respectively) than grain morphology traits, such as grain length (GL), grain width (GW), and length-to-width ratio (LWR). These results demonstrate that the panel possessed sufficient phenotypic diversity to support subsequent genomic prediction analyses.

### 3.2. Genome-Wide Distribution of SNP Markers

After quality control, a total of 4647 high-quality SNP markers were retained for subsequent analyses. These markers were distributed across all 12 rice chromosomes with relatively uniform genome-wide coverage (Figure 1). The target regions from which the SNPs were designed originated from three complementary sources, including genome-wide association studies (GWASs), candidate genes, and selective sweep analysis (SA), providing broad representation of genomic regions associated with important agronomic traits. The filtered SNP dataset was subsequently used for population structure analysis, genome-wide association analysis, genomic prediction, and training population optimization.

### 3.3. Population Genetic Structure Analysis

PCA revealed substantial genetic variation among the 829 rice accessions. PC1, PC2, and PC3 explained 39.78%, 20.44%, and 11.09% of the total genetic variation, respectively. The PCA plots showed that subgroup C was relatively separated from the other accessions along PC1, whereas subgroups A and B exhibited considerable overlap. Additional differentiation among the accessions was observed along PC2 and PC3 (Figure 2).

The elbow analysis of K-means clustering indicated that K = 3 provided a suitable operational grouping of the accessions, as the reduction in within-cluster sum of squares became less pronounced after three clusters (Supplementary Figure S2). The three resulting groups contained 314, 317, and 198 individuals and were designated as subgroups A, B, and C, respectively. The PCA distribution and phylogenetic tree distributions indicated that these groups captured major patterns of genetic differentiation in the panel, although substantial overlap remained between some groups, particularly A and B (Figure 3).

The three groups were subsequently used as operational genetic subgroups for cross-population prediction. Thus, the K-means classification was used to define contrasting genetic backgrounds for prediction-transferability analysis rather than to infer the existence of three biologically discrete populations.

### 3.4. Comparison of Genomic Selection Models in the Full Population

To evaluate the predictive performance of different genomic selection (GS) models, repeated five-fold cross-validation was performed using the full population of 829 rice accessions. The predictive performance of 17 GS models was compared across eight agronomic traits, and predictive performance was evaluated using the Pearson correlation coefficient (*r*) between observed and predicted phenotypic values. The mean predictive performance and associated variability across repeated cross-validation analyses are presented in Figure 4 and Supplementary Figure S1.

Predictive performance varied substantially among traits and models. Grain-related traits, including grain length (GL), grain width (GW), length-to-width ratio (LWR), thousand-grain weight (TGW), and heading date (HD), generally showed relatively high predictive performance, whereas grain number per panicle (GN) and panicle number (PN) showed moderate values. Seed-setting rate (SR) exhibited the lowest predictive performance among the eight traits. These differences indicate that the predictive performance of GS models was strongly trait-dependent.

Overall, no single GS model consistently showed the highest observed predictive performance across all traits. EnsembleGS showed the highest observed predictive performance for GL, GW, LWR, and TGW, whereas RKHS showed the highest observed predictive performance for GN and PN, and RFR showed the highest observed predictive performance for HD. RR-BLUP and RFR showed similar predictive performance for SR. This trait-specific variation indicates that model performance depended on the characteristics of individual traits. Several models showed similar predictive performance within individual traits, with overlapping uncertainty intervals, indicating that relatively small differences in mean predictive performance should not be interpreted as evidence of clear differences among models.

To provide a descriptive summary of model performance across traits, the mean predictive performance across the eight agronomic traits was calculated for each GS model (Figure 5). These averages are presented solely as descriptive summaries of predictive performance across traits and were not used to make an overall ranking of the evaluated models. The cross-trait averages should therefore be interpreted with caution because the eight traits differ in their genetic architecture, phenotypic variation, and inherent predictability. Accordingly, model performance was primarily evaluated on a trait-specific basis rather than according to the eight-trait average.

### 3.5. Effects of Different Prediction Strategies on Genomic Selection Performance

#### 3.5.1. Distribution and Stability of STPGA-Selected Training Populations

To evaluate the genetic representativeness and stability of STPGA-selected training populations, independent STPGA optimization was performed using 10 different random seeds for each training population size of 100, 200, 300, 400, and 500 individuals. For each run, the selected individuals were projected onto the principal component (PC) space derived from the complete set of 829 rice accessions and were also examined in the corresponding phylogenetic tree. The same PCA and phylogenetic tree were used across all independent STPGA runs because the underlying genotype dataset remained unchanged.

For a training population size of 100 individuals, the STPGA-selected individuals were distributed across the genetic variation represented in the complete panel (Figure 6). Similar overall distributions were observed across the independent runs, although the specific individuals selected varied among runs. As the training population size increased from 100 to 500 individuals, the selected populations represented an increasing number of accessions from the complete panel and were distributed across the major regions of the genetic structure. The PCA and phylogenetic tree distributions indicated that STPGA selections were not restricted to a single genetic subgroup or a small number of closely related individuals (Figure 7).

Variation in the specific individuals selected among independent runs was also observed, reflecting the stochastic nature of the genetic algorithm. The 10 independent runs therefore provided an assessment of between-run variation in STPGA selection. The PCA and phylogenetic distributions of the independent STPGA runs for each training population size are presented in Supplementary Figures S2 and S3.

For each training population size, the accessions not selected by STPGA constituted the corresponding prediction target. Thus, the size and composition of the prediction target differed among training population sizes. The subsequent prediction analyses evaluated how the selected training populations performed in predicting their corresponding remaining accessions.

#### 3.5.2. Comparison of Different Prediction Strategies

The predictive performance of genomic selection was compared among full-population five-fold cross-validation, cross-population prediction, and STPGA-optimized training populations of different sizes. For full-population cross-validation, all 829 accessions were included in repeated five-fold cross-validation. For cross-population prediction, the three K-means-defined subgroups contained 314, 317, and 198 individuals, respectively, and were evaluated using the A → BC, B → AC, and C → AB prediction schemes.

Across the three prediction strategies, full-population five-fold cross-validation generally produced the highest predictive performance. Cross-population prediction showed lower performance for most traits and prediction scenarios, although the magnitude of the reduction varied among traits and among the A → BC, B → AC, and C → AB schemes. The relative performance of individual prediction models also changed across transfer scenarios, indicating that model performance depended on both trait and training-to-testing population configuration. Because the three subgroups differed in sample size and because the training and prediction target populations differed among the three prediction scenarios, the cross-population results were interpreted as an assessment of prediction transferability across genetically differentiated populations rather than as a strictly sample-size-controlled comparison of predictive performance.

For the STPGA analysis, training populations of 100, 200, 300, 400, and 500 individuals were selected, and the corresponding remaining accessions were used as prediction targets. Thus, both training population size and prediction target size varied across the evaluated training population sizes. Predictive performance under STPGA varied with both trait and training population size and did not show a uniformly monotonic increase as the training population became larger. For GL, for example, the highest predictive performance was observed with the 400-individual training population (RFR, *r* = 0.800), whereas the performance at 500 individuals was slightly lower for most models (Figure 8). For GN, the highest performance was observed at 300 individuals (RKHS, *r* = 0.635), while for LWR the highest performance was also observed at 300 individuals (RFR, *r* = 0.816). In contrast, PN and TGW showed their highest values at 500 individuals, reaching *r* = 0.745 and 0.803, respectively. These results indicate that the relationship between training population size and predictive performance was trait-dependent rather than uniformly increasing with additional training individuals. Detailed results for the other seven traits are presented in Supplementary Figure S4.

Compared with full-population five-fold cross-validation, the STPGA-selected training populations generally achieved lower predictive performance but retained substantial predictive performance with considerably smaller training populations. The extent to which predictive performance was retained varied among traits and models. These results demonstrate that STPGA can identify informative subsets of individuals for genomic prediction, while the appropriate training population size may depend on the target trait and the desired balance between training population size and predictive performance.

Overall, the three prediction strategies revealed complementary aspects of genomic prediction. Full-population cross-validation characterized model performance when the training and testing samples were drawn from the same overall population; cross-population prediction evaluated the transferability of prediction models between genetically differentiated subgroups; and STPGA evaluated how predictive performance changed when informative training subsets of different sizes were used. Accordingly, these analyses were interpreted separately rather than combined into a single overall ranking of prediction strategies or prediction models.

#### 3.5.3. Comparison of STPGA and Random Sampling

To evaluate whether STPGA provided an advantage over random selection at comparable training population sizes, STPGA and random sampling were compared using training populations of 100, 200, 300, 400, and 500 individuals. STPGA was independently repeated 10 times using different random seeds for each training population size, whereas random sampling was independently repeated 100 times. In both approaches, training populations were selected from the full set of 829 rice accessions, and the remaining accessions were used as the corresponding prediction target.

For GL, the mean prediction performance of the STPGA-selected training populations was generally higher than that obtained from random sampling across the evaluated training population sizes. Under both sampling strategies, prediction performance tended to improve as the training population size increased, although the magnitude and consistency of improvement varied among training population sizes. The standard deviation across the 10 independent STPGA runs was used to quantify variation in prediction performance associated with the stochastic optimization procedure (Figure 9).

Similar patterns were observed for several other traits, although the difference between STPGA and random sampling varied among traits and training population sizes. For traits with relatively low prediction performance, such as SR, the difference between the two sampling strategies was generally smaller. These results indicate that the benefit of STPGA was trait-dependent rather than uniform across all traits. Detailed comparisons for the other seven traits are presented in Supplementary Figure S5.

Overall, the repeated analyses showed that STPGA-selected training populations generally achieved higher mean prediction performance than randomly selected training populations at comparable training population sizes for several traits. However, the magnitude of the difference varied across traits and training population sizes. Because the corresponding prediction target changed with training population size, these comparisons were interpreted as comparisons between STPGA and random sampling under the same training population size and sampling framework, rather than as a strictly controlled assessment of the effect of training population size using a fixed prediction target. Detailed prediction performance, standard deviations, and results for all traits and training population sizes are provided in Supplementary Table S4 and Figure S5.

### 3.6. GWAS Analysis and Genomic Prediction Using GWAS-Prioritized SNP Subsets

#### 3.6.1. GWAS of the Eight Agronomic Traits

Among the evaluated prediction strategies, the STPGA-optimized training population consisting of 400 individuals was selected for subsequent genome-wide association analysis (GWAS) to provide a consistent training population for marker prioritization. This training population represented a practical compromise between training population size and predictive performance within the STPGA analysis.

GWAS was performed for the eight agronomic traits using the BLINK method. Manhattan plots were generated using a nominal threshold of *p* < 10^−5^ to highlight relatively strong or suggestive association signals, and multiple-testing correction was not applied because the GWAS results were used for marker prioritization rather than formal hypothesis testing (Figure 10a). The corresponding quantile–quantile (Q–Q) plots showed that the observed *p*-value distributions generally followed the expected distributions, with deviations primarily observed among the most strongly associated loci (Figure 10b).

#### 3.6.2. Genomic Prediction Using GWAS-Prioritized and Random SNP Subsets

To evaluate whether GWAS-guided marker prioritization could retain predictive information more efficiently than random marker reduction, genomic prediction was performed using five marker-set conditions: the complete 4647-SNP dataset (Full4647), GWAS-prioritized Top1000 and Top500 SNP subsets (GWAS-Top1000 and GWAS-Top500), and randomly selected 1000- and 500-SNP subsets (Random1000 and Random500). The random subsets were independently generated 100 times for each marker density. All marker sets were evaluated using the same genomic prediction framework, and prediction performance was assessed using Pearson’s correlation coefficient (*r*).

The Full4647 dataset provided the complete-marker reference, whereas the Random1000 and Random500 subsets served as marker-density-matched controls for evaluating the effect of GWAS-based marker prioritization. Thus, comparisons between GWAS-prioritized and randomly selected subsets at the same marker density were used to distinguish the effect of marker prioritization from that of marker reduction alone. Prediction performance for the random subsets was summarized across the 100 independent replicates, whereas the GWAS-prioritized subsets represented fixed marker sets.

The effect of GWAS-based marker prioritization varied substantially among traits and marker densities. For GL, GWAS-prioritized subsets generally showed higher prediction performance than the corresponding random subsets. For example, the highest model-specific performance reached 0.789 for GWAS-Top500 compared to 0.751 for Random500, and 0.802 for GWAS-Top1000 compared to 0.775 for Random1000. A similar pattern was observed for LWR, for which the highest performance increased from 0.727 for Random500 to 0.764 for GWAS-Top500 and from 0.783 for Random1000 to 0.800 for GWAS-Top1000. GW also showed a modest advantage of GWAS-prioritized markers at both marker densities, although the magnitude of the difference was smaller than that observed for GL and LWR. The corresponding results for the other traits are presented in Supplementary Figure S6.

In contrast, the GWAS-prioritized subsets did not consistently outperform random subsets for the other traits. For GN, HD, PN, and SR, several models showed higher prediction performance using Random500 or Random1000 than using the corresponding GWAS-prioritized subsets. For TGW, the differences between GWAS-prioritized and random subsets were generally small, and the random subsets slightly exceeded the GWAS-prioritized subsets for several models. These results indicate that the predictive value of GWAS-prioritized markers was trait-dependent and that GWAS-based marker selection did not universally improve genomic prediction at a given marker density.

Comparison with the STPGA-selected 400-individual training population using the complete 4647-SNP dataset provided an additional reference for evaluating the extent of information retained after marker reduction. Under the STPGA-optimized 400-individual training population, the highest observed predictive performance across models reached approximately 0.800 for GL, 0.623 for GN, 0.775 for GW, 0.761 for HD, 0.799 for LWR, 0.722 for PN, 0.452 for SR, and 0.787 for TGW. Relative to these full-marker STPGA benchmarks, the 1000-SNP subsets retained a substantial proportion of predictive performance for most traits. The reduction to 500 SNPs resulted in a larger loss of performance for several traits, although the magnitude of this loss differed among traits. Notably, for GL and LWR, the GWAS-Top1000 subsets reached values comparable to or slightly above the corresponding STPGA-400 full-marker benchmark for models showing the highest observed predictive performance, whereas the other traits generally showed lower performance after marker reduction.

The comparison therefore suggests that reducing the marker set from 4647 to 1000 SNPs can retain a substantial amount of genomic prediction information, but the extent of information retention depends on the trait. GWAS-based prioritization appeared particularly useful for some traits, such as GL and LWR, but did not provide a consistent advantage across all eight traits. Importantly, these results should be interpreted as a comparison of marker-selection strategies rather than as evidence that a reduced GWAS-prioritized marker set universally improves prediction performance. The results also demonstrate that marker density and marker prioritization represent partially independent factors affecting genomic prediction performance. Detailed numerical results for all eight traits and the 17 genomic selection models are provided in Supplementary Table S6, with graphical comparisons shown in Figure 11 and Supplementary Figure S6.

## 4. Discussion

### 4.1. Limitations of Single-Environment Phenotypic Data

The present study was based on phenotypic data collected from a single field environment and therefore did not explicitly account for genotype-by-environment (G × E) interactions. Consequently, the prediction performance reported here primarily reflects model performance under the environmental conditions represented by the current experiment and should not be directly interpreted as the expected performance across diverse environments. Previous studies have demonstrated that G × E interactions can substantially influence genomic prediction, particularly for complex agronomic traits, and that multi-environment genomic prediction can provide a more realistic assessment of breeding performance across environments [3].

An additional limitation is that the phenotypic evaluation was conducted without independent field replications, blocking, or randomized allocation of accessions. The phenotypic values were therefore based on accession-level measurements from a single field evaluation rather than replicated estimates such as BLUEs or BLUPs. Consequently, field-level and plant-level environmental variation could not be fully separated from genetic effects or reduced through replicated experimental design. Such phenotypic noise may have reduced the reliability of the observed phenotypic values and may therefore underestimate the attainable prediction performance that could be achieved using more precise estimates of genetic value. In addition, because variance components could not be reliably estimated from the present experimental design, heritability was not estimated in this study.

These limitations should be considered when interpreting the reported prediction performance. Nevertheless, the common phenotypic dataset provided a consistent basis for comparing prediction models, population structures, training population designs, and marker-selection strategies within the same field environment. The present results should therefore be regarded as an initial evaluation under a single environmental condition rather than as a definitive assessment of genomic selection performance across environments or breeding cycles. Future studies should incorporate multi-environment trials with independent field replications, appropriate blocking and randomization, and spatial adjustment where applicable, together with models that explicitly account for G × E interactions. Such designs will be important for obtaining more precise estimates of genetic values and for validating the robustness and practical applicability of genomic selection across diverse rice-growing environments.

### 4.2. Genomic Selection Under Low-Density Targeted Sequencing

Targeted sequencing provides an economical alternative to whole-genome genotyping, but reduced marker density raises concerns regarding the amount of genomic information available for prediction. In the present study, substantial prediction performance was obtained using only 4647 SNP markers, demonstrating that a breeding-oriented targeted sequencing panel can provide sufficient marker information for genomic prediction across a range of rice agronomic traits.

Prediction performance varied substantially among traits. Grain morphology-related traits, including GL, GW, LWR, and TGW, generally showed higher prediction performance than traits such as SR and GN. This pattern is consistent with previous studies showing that traits with relatively higher heritability or simpler genetic architectures are generally more amenable to genomic prediction than complex traits strongly affected by environmental variation and numerous loci with small effects [26,27].

The relative performance of prediction models also varied among traits. In the full-population repeated five-fold cross-validation, EnsembleGS showed the highest prediction performance for GL, GW, LWR, and TGW, whereas RKHS performed best for GN and PN, RFR performed best for HD, and RR-BLUP and RFR showed comparable performance for SR. Thus, no single model consistently achieved the highest prediction performance across all eight traits. These results indicate that model performance is trait-dependent rather than determined solely by the broad distinction between linear, Bayesian, machine-learning, or deep-learning approaches. In particular, the results do not support a universal advantage of one model category over the others under the present targeted-sequencing framework [1]. RR-BLUP showed variable performance across traits rather than a consistent rank among the 17 models, with relatively lower prediction performance for some traits, including GL, compared with models such as EnsembleGS and RKHS. Notably, EnsembleGS showed relatively high prediction performance across several traits in the present study, with Pearson correlation coefficients of 0.838 for GL, 0.832 for GW, 0.844 for LWR, and 0.818 for TGW. The relatively strong performance of EnsembleGS may be related to its combination of complementary prediction models, including RR-BLUP, BRR, LightGBM, MLPGS, and DeepBLUP. These component models differ in their assumptions about marker effects and genotype–phenotype relationships, and their combination may potentially capture complementary components of genetic signal. Ensemble approaches have also been explored in recent genomic prediction studies as a strategy for integrating predictions from models with different statistical and learning assumptions. However, the present results do not demonstrate that ensemble modeling consistently provides higher predictive performance than individual algorithms, because RKHS, RFR, and RR-BLUP or RFR showed the highest or similar predictive performance for several other traits. Therefore, the relatively high performance of EnsembleGS should be interpreted as evidence that combining complementary prediction approaches can be useful under the present targeted-sequencing framework, rather than as evidence for a universally preferred prediction algorithm.

An additional limitation is that the model comparison was performed using predefined and fixed parameter settings rather than extensive model-specific hyperparameter optimization. This standardized strategy facilitated a consistent comparison across the 17 models and ensured that differences in model-specific tuning procedures did not affect the evaluation framework. However, models that are more sensitive to hyperparameter configuration may not have reached their maximum attainable predictive performance under the evaluated settings. Therefore, the relative performance observed in this study should be interpreted within the predefined parameter framework and should not be regarded as evidence that any particular model is intrinsically preferable to the others.

Overall, these findings indicate that genomic prediction using a breeding-oriented targeted sequencing panel can achieve substantial prediction performance with a relatively limited number of SNP markers. The results further suggest that marker density alone does not determine genomic prediction performance; the choice of prediction model and the genetic architecture of the target trait also contribute to variation in predictive performance. Rather than relying exclusively on increasing marker density, combining a carefully designed marker panel with complementary prediction algorithms may provide a practical strategy for genomic selection in rice breeding programs. This approach may be particularly relevant for routine breeding programs in which a large number of breeding materials need to be genotyped and evaluated [28,29].

### 4.3. Population Structure Influences Genomic Prediction Performance

Population structure is an important factor influencing genomic prediction because prediction performance depends partly on the genetic relationships between the training and target populations [30]. In the present study, principal component and phylogenetic analyses revealed genetic differentiation among the 829 rice accessions. Consistent with this structure, cross-population prediction generally showed lower prediction performance than full-population five-fold cross-validation, although the magnitude of the reduction varied among traits and transfer directions.

The reduction in cross-population prediction performance may reflect differences in allele frequencies, linkage disequilibrium patterns, and genomic relationships between training and target populations, which can reduce the transferability of marker effects across genetically differentiated groups [31,32]. Similar reductions in prediction performance under cross-population prediction have been reported in rice, maize, wheat, and other crops [33]. However, the three transfer scenarios evaluated here involved different subgroup sizes and different training and target populations. Therefore, the A → BC, B → AC, and C → AB results should be interpreted primarily as assessments of prediction transferability across genetically differentiated populations rather than as strictly sample-size-controlled comparisons.

These findings emphasize the importance of constructing training populations that adequately represent the genetic diversity of the target breeding population. In practical genomic selection programs, a training population that is genetically representative of the breeding materials to which predictions will be applied may be more informative than a training population selected solely on the basis of numerical sample size.

### 4.4. Training Population Optimization and Prediction Performance

The composition and size of the training population are important determinants of genomic prediction performance. STPGA provides a strategy for selecting individuals that are informative for the prediction target by optimizing the composition of the training population with respect to prediction error variance and genetic representativeness [10].

In the present study, STPGA-selected training populations of 100, 200, 300, 400, and 500 individuals were evaluated. Prediction performance generally improved from the smallest training populations toward intermediate or larger training population sizes for several traits, but the response was not uniformly monotonic and differed among traits and prediction models. The training population size associated with the highest observed prediction performance varied among traits. For example, the highest performance for GL was observed at 400 individuals, whereas GN, GW, LWR, and SR reached their highest values at 300 individuals. In contrast, PN and TGW showed their highest values at 500 individuals.

These results indicate that increasing the number of training individuals does not necessarily produce a proportional or monotonic improvement in prediction performance. Instead, the relationship between training population size and prediction performance depends on the trait, model, and composition of the selected training population. Importantly, the model that performed best in full-population cross-validation did not necessarily remain optimal after STPGA-based training population reduction. This finding highlights the importance of jointly considering training population composition, training population size, and model choice.

The STPGA results also demonstrate that substantial prediction performance can be retained using considerably smaller optimized training populations than the full set of 829 accessions. This has practical implications for breeding programs in which phenotyping resources are limited. By preferentially selecting genetically informative individuals, training population optimization may reduce the number of individuals requiring extensive phenotyping while maintaining useful predictive performance [11]. Nevertheless, the most appropriate training population size should be determined according to the target trait and breeding objective rather than assuming that a single training population size is universally preferable.

The comparison between STPGA-selected and randomly selected training populations should also be interpreted with caution because the two sets were not evaluated as strictly paired training populations. Random training populations were generated independently, whereas STPGA-selected populations were constructed through an optimization procedure based on genetic information. Therefore, the observed differences in predictive performance reflect the outcomes of the respective sampling strategies under the implemented analysis scheme, rather than a strictly paired statistical comparison in which matched training populations differed only in their selection strategy. Accordingly, these results provide an empirical reference for evaluating training population selection strategies, but do not by themselves establish a universal advantage of STPGA over random sampling.

### 4.5. Integrating GWAS with Genomic Selection

An important objective of this study was to determine whether GWAS-guided marker prioritization could retain predictive information after reducing marker density. The results showed that the effect of GWAS-based marker prioritization was trait- and marker-density-dependent. At both 500- and 1000-SNP densities, GWAS-prioritized subsets showed clear advantages over corresponding random subsets for some traits, particularly GL and LWR. For other traits, however, random subsets achieved similar or higher prediction performance than GWAS-prioritized subsets. Therefore, the present results do not support a universal advantage of GWAS-based marker prioritization across all traits.

The comparison between GWAS-prioritized and random marker subsets provides an important distinction between marker reduction and marker prioritization. Because Random500 and Random1000 were independently sampled at the same marker densities as GWAS-Top500 and GWAS-Top1000, differences between the paired conditions provide a direct assessment of whether trait-informed marker selection retained additional predictive information beyond marker reduction alone. The results indicate that this additional benefit depends on the genetic architecture of the target trait and the extent to which GWAS-prioritized markers capture relevant genetic variation.

Comparison with the STPGA-selected 400-individual training population using the complete 4647-SNP dataset further showed that reducing the marker set to 1000 SNPs could retain substantial predictive performance for several traits. The extent of information retained varied among traits, and further reduction to 500 SNPs resulted in larger performance changes for some traits. These results suggest that marker density and marker prioritization should be considered as separate but interacting components of genomic prediction design.

The observation that some reduced marker sets slightly exceeded the corresponding full-marker reference for individual traits or models should also be interpreted cautiously. Such differences may arise from model-specific variation, sampling effects, or the removal of less informative markers, rather than indicating that fewer markers are intrinsically more effective than a complete marker set. Accordingly, the main implication of the present analysis is not that GWAS-based reduction universally improves prediction, but that trait-informed marker prioritization can provide a useful strategy for retaining predictive information under reduced marker density.

The integration of GWAS with targeted sequencing therefore provides a potentially useful framework for developing cost-efficient genomic prediction systems. However, the magnitude and consistency of the benefit should be evaluated separately for each target trait rather than assumed to be universal across traits.

### 4.6. Implications for Rice Breeding and Future Perspectives

The present study demonstrates that breeding-oriented targeted sequencing can provide substantial genomic prediction performance when combined with appropriate prediction models, genetically informed training population design, and trait-informed marker prioritization. Rather than relying solely on increasing marker density or training population size, the results indicate that complementary optimization strategies can help balance genomic information, prediction performance, and operational cost.

From a practical breeding perspective, the results provide several considerations for implementing genomic selection in rice breeding programs. First, a targeted sequencing panel containing 4647 SNPs provided substantial predictive information across multiple agronomic traits, supporting its potential use as a cost-efficient genotyping platform. Second, prediction performance was strongly influenced by population composition, emphasizing the importance of matching the training population to the genetic background of the target breeding materials. Third, STPGA demonstrated that informative subsets of individuals can retain useful prediction performance, although the appropriate training population size varied among traits. Finally, GWAS-prioritized marker subsets provided an efficient alternative to random marker reduction for some traits, but their benefit was not universal and should therefore be evaluated in a trait-specific manner.

Taken together, these findings support an integrated framework in which marker density, marker informativeness, training population composition, and prediction model selection are considered jointly rather than optimized independently. Such a framework may be particularly useful for breeding programs operating under constraints on genotyping and phenotyping resources.

Future studies should incorporate multi-environment phenotypic data, larger and more genetically diverse training populations, and additional molecular information, including transcriptomic and metabolomic data, to further evaluate prediction robustness [34]. Independent validation in breeding populations not included in model development will also be important for determining the transferability of the proposed framework. In addition, validation across multiple breeding cycles and environments will provide stronger evidence for its practical utility in genomic-assisted rice.

## 5. Conclusions

This study systematically evaluated the prediction performance of 17 genomic selection (GS) models using 4647 SNP markers generated by targeted sequencing and investigated the effects of population structure, training population design, and GWAS-guided marker prioritization on genomic prediction in rice.

The 4647-SNP targeted sequencing panel provided substantial predictive information across the eight agronomic traits evaluated. Prediction performance varied among traits and GS models, and no single model consistently achieved the highest performance across all traits. Population structure also had an important influence on prediction transferability, with cross-population prediction generally showing lower performance than full-population five-fold cross-validation. The magnitude of this reduction varied among traits and training-to-testing population configurations.

STPGA-based training population optimization demonstrated that useful prediction performance could be retained using substantially smaller training populations. However, the relationship between training population size and prediction performance was not uniformly monotonic and differed among traits and models. The training population size associated with the highest observed performance therefore depended on the target trait, highlighting the importance of jointly considering training population composition and size rather than relying on a single population size for all traits.

GWAS-guided marker prioritization also showed a trait-dependent effect. For some traits, particularly GL and LWR, GWAS-prioritized 500- and 1000-SNP subsets showed higher prediction performance than marker-density-matched random subsets, whereas other traits showed similar or higher performance with randomly selected markers. These results indicate that GWAS-guided marker reduction can retain substantial predictive information for selected traits, but its advantage over random marker reduction is not universal.

Overall, the present study supports an integrated framework combining breeding-oriented targeted sequencing, trait- and population-informed training population design, and GWAS-guided marker prioritization for cost-efficient genomic selection in rice. Rather than relying on a single optimization strategy, jointly considering marker density, marker informativeness, training population composition, training population size, and prediction model selection provides a practical framework for balancing prediction performance with genotyping and phenotyping resources. Further validation using multi-environment trials and independent breeding populations will be important for determining the robustness and practical applicability of this framework in rice breeding programs.

## Figures and Tables

**Figure 1:**
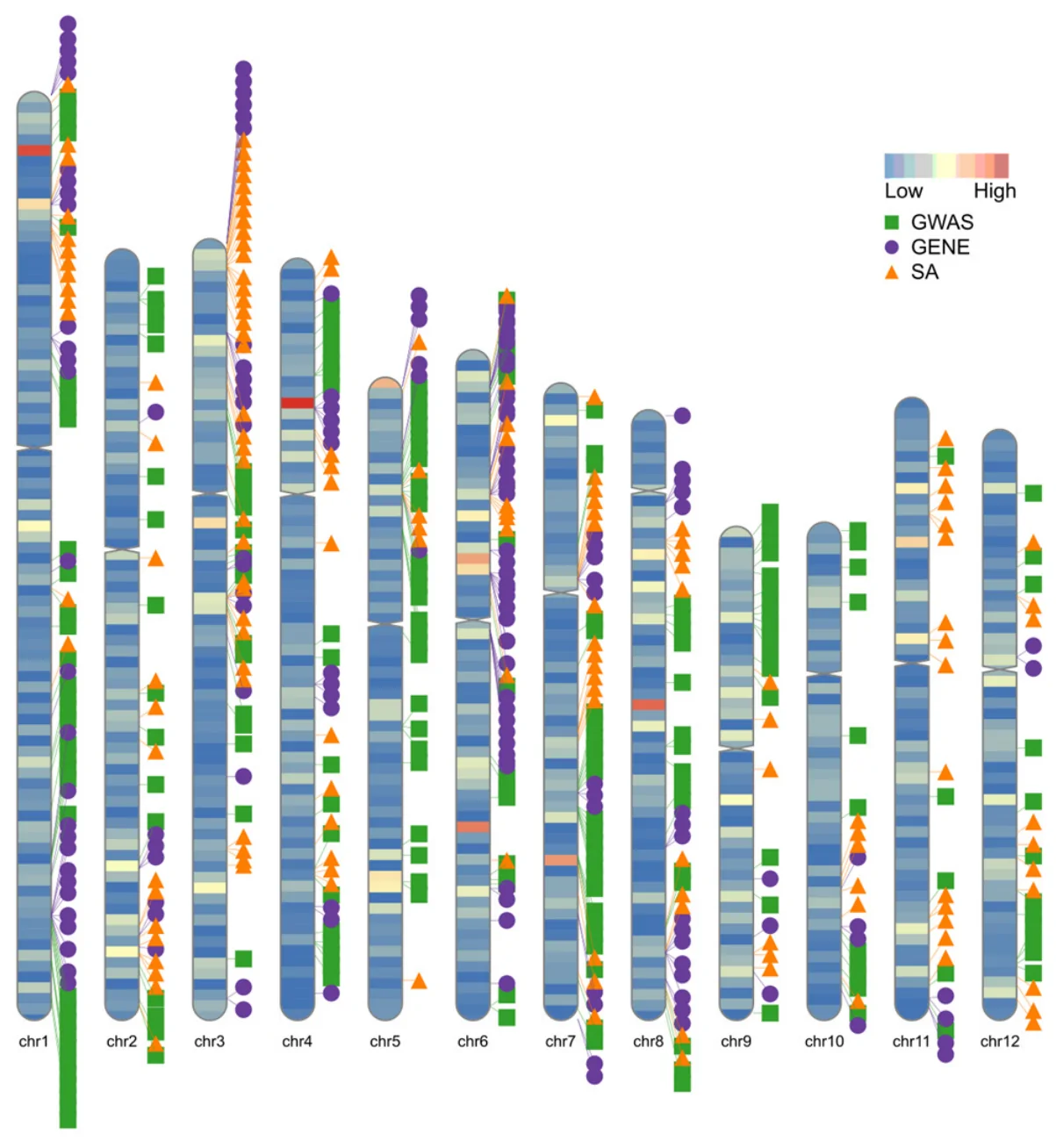
Chromosomal distribution and density of targeted SNPs across the 12 rice chromosomes. The sources of target regions used for panel design, including genome-wide association studies (GWASs), candidate genes, and selective sweep analysis (SA), are indicated alongside each chromosome.

**Figure 2:**
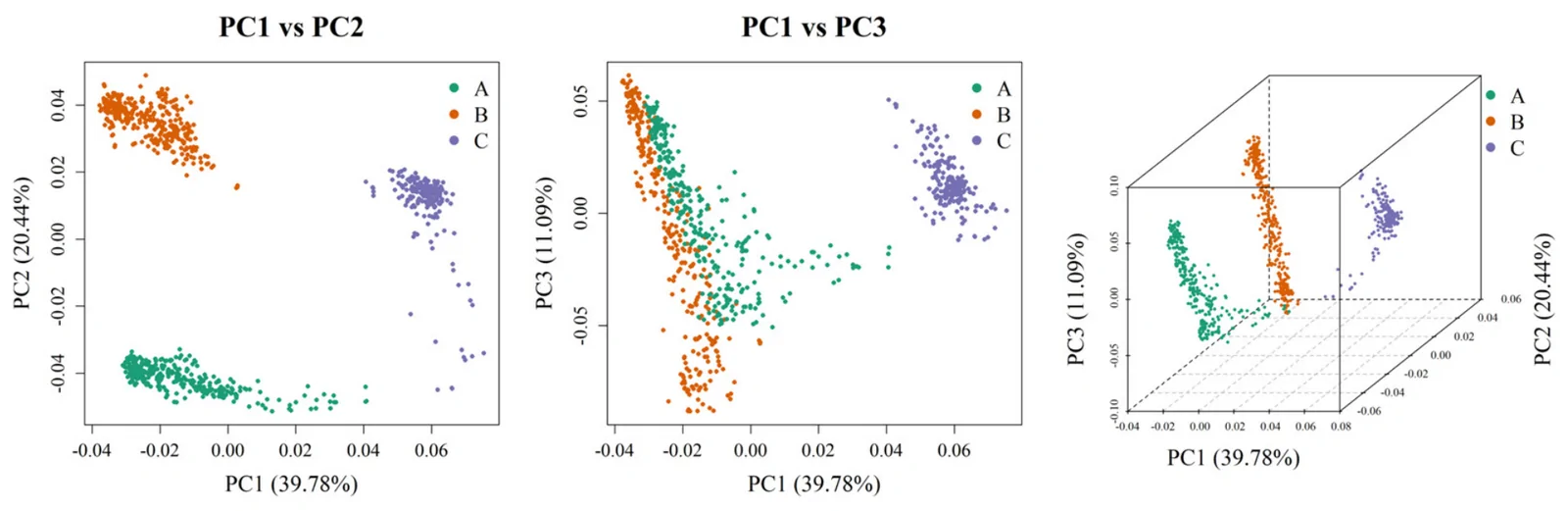
Principal component analysis (PCA) showing the genetic structure of the 829 rice accessions based on genome-wide SNP markers.

**Figure 3:**
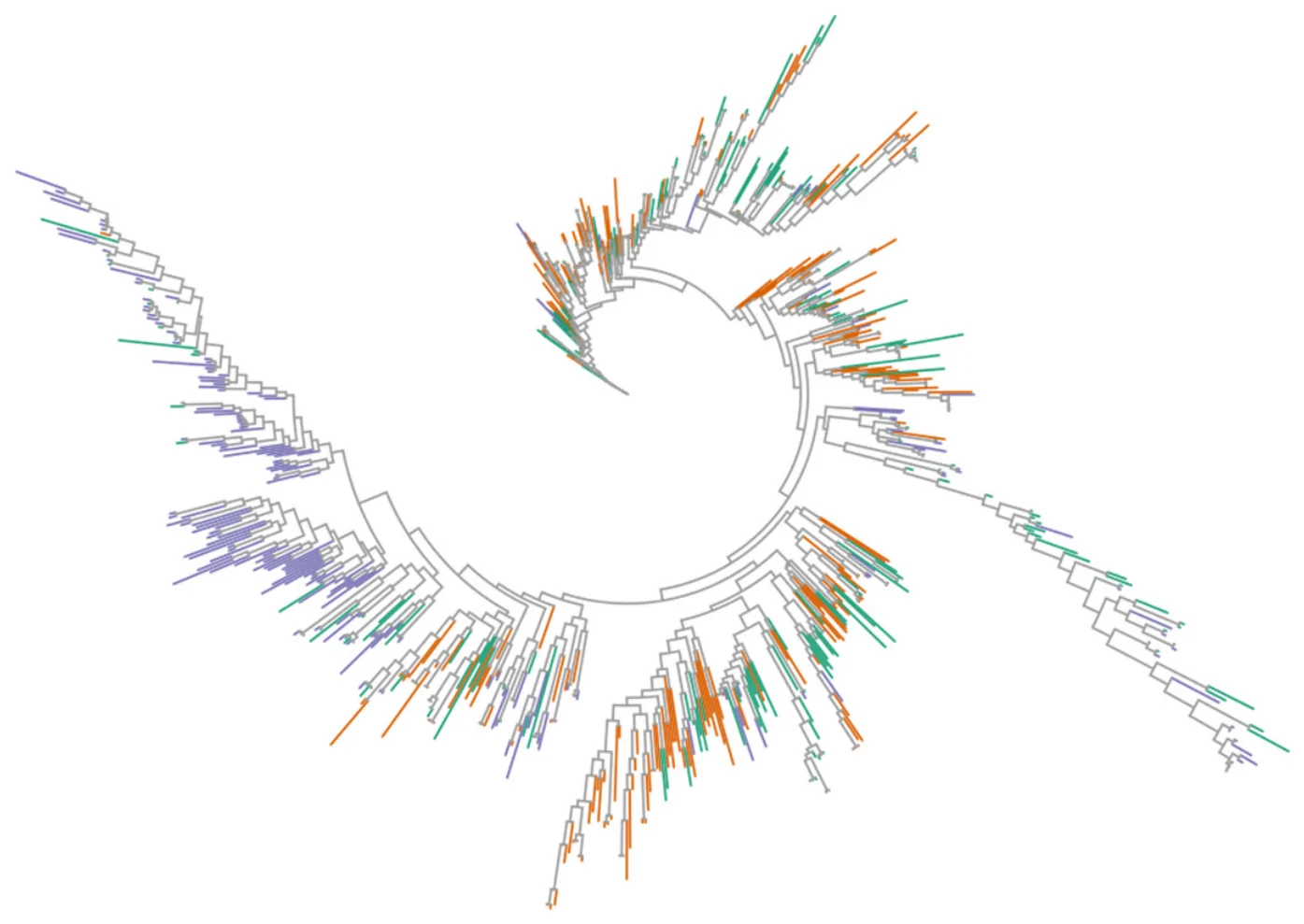
Phylogenetic tree showing the genetic relationships among the 829 rice accessions based on genome-wide SNP markers, colors correspond to the three genetic groups defined in Figure 2: green = group A, orange = group B, blue = group C.

**Figure 4:**
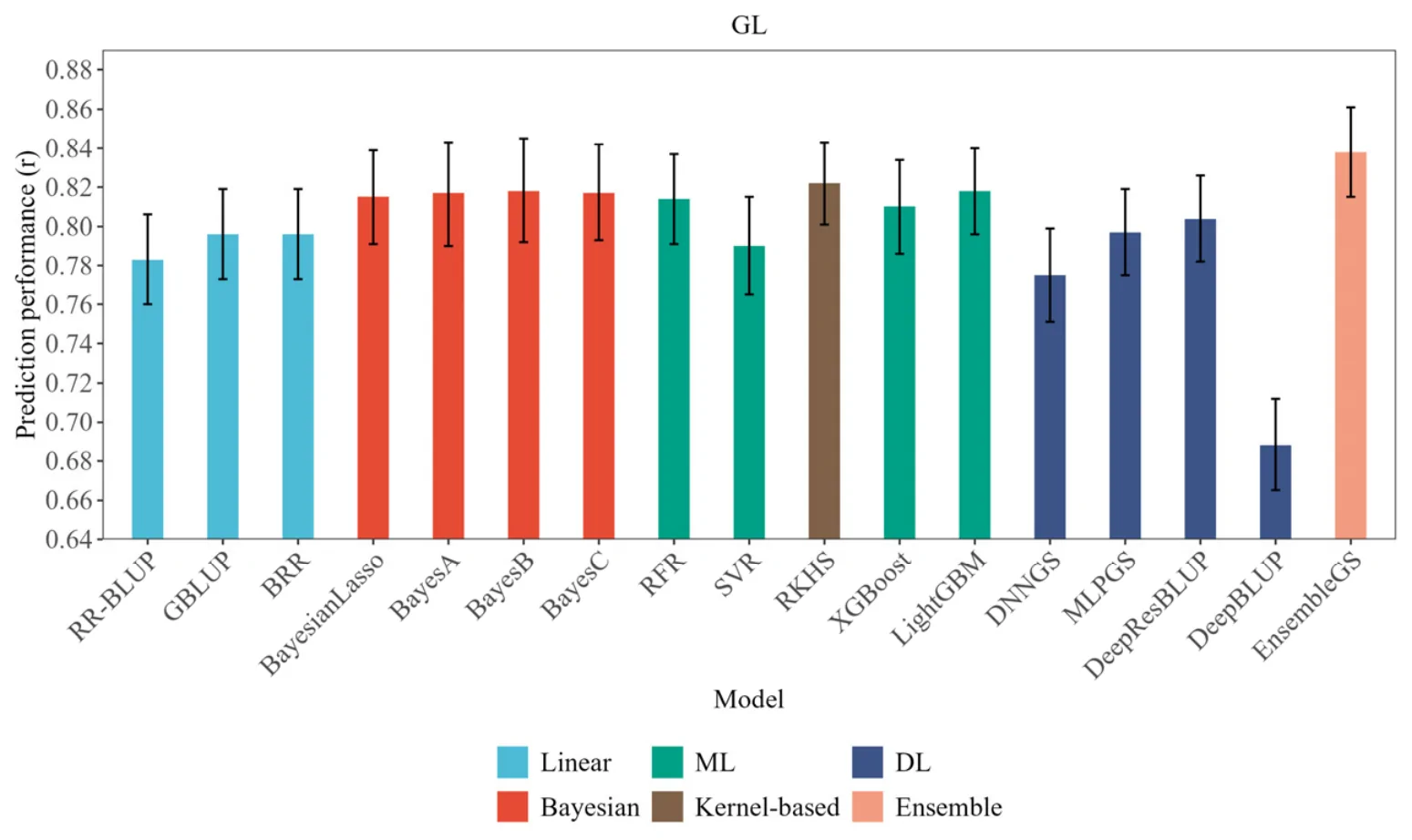
Comparison of prediction performance among 17 genomic selection models for grain length (GL) based on repeated five-fold cross-validation in the full population. Bars represent mean Pearson correlation coefficients (*r*), and error bars indicate the corresponding 95% confidence intervals across repeated cross-validation analyses.

**Figure 5:**
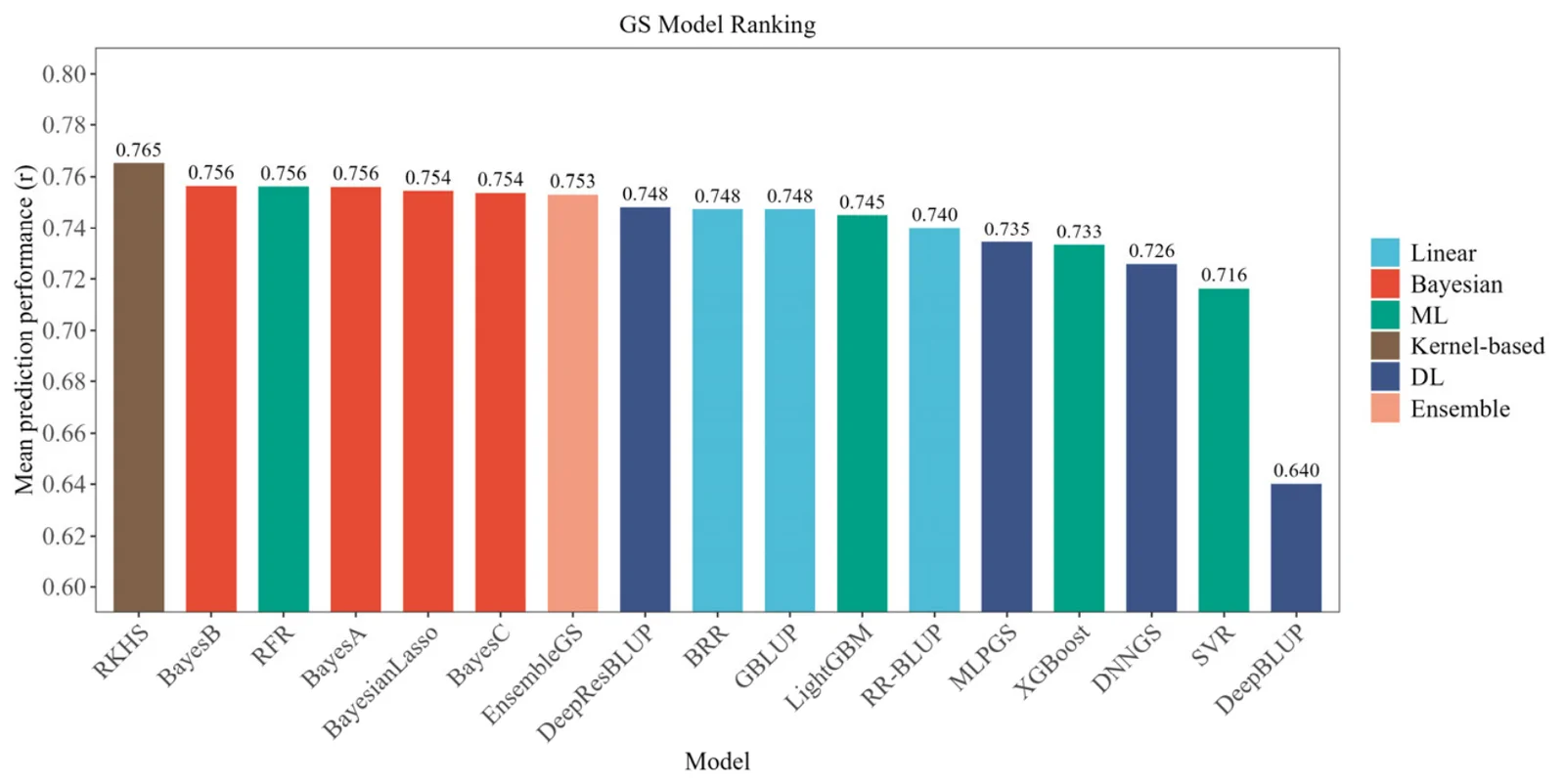
Descriptive comparison of the mean prediction performance of the 17 genomic selection models across the eight agronomic traits. Cross-trait means are presented as descriptive summaries and are not used to define an overall best-performing model.

**Figure 6:**
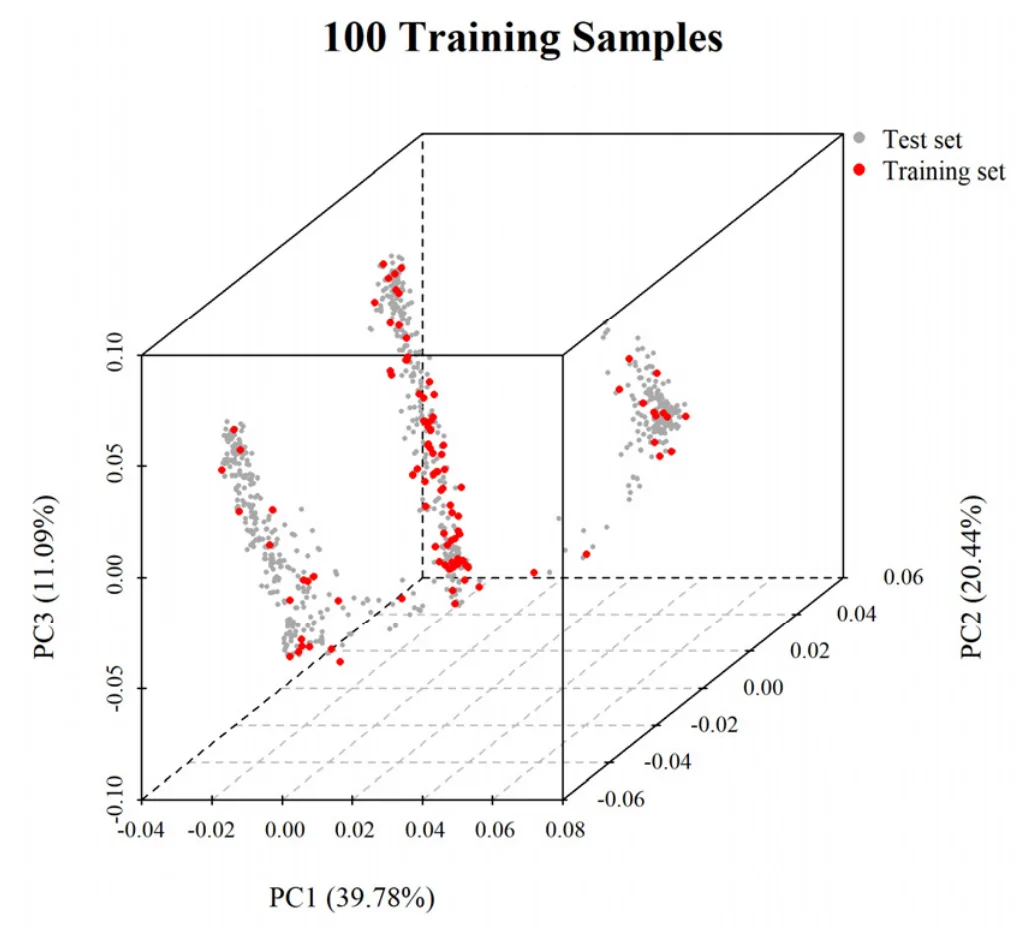
Distribution of the STPGA-selected training population of 100 individuals in the principal component (PCA) space.

**Figure 7:**
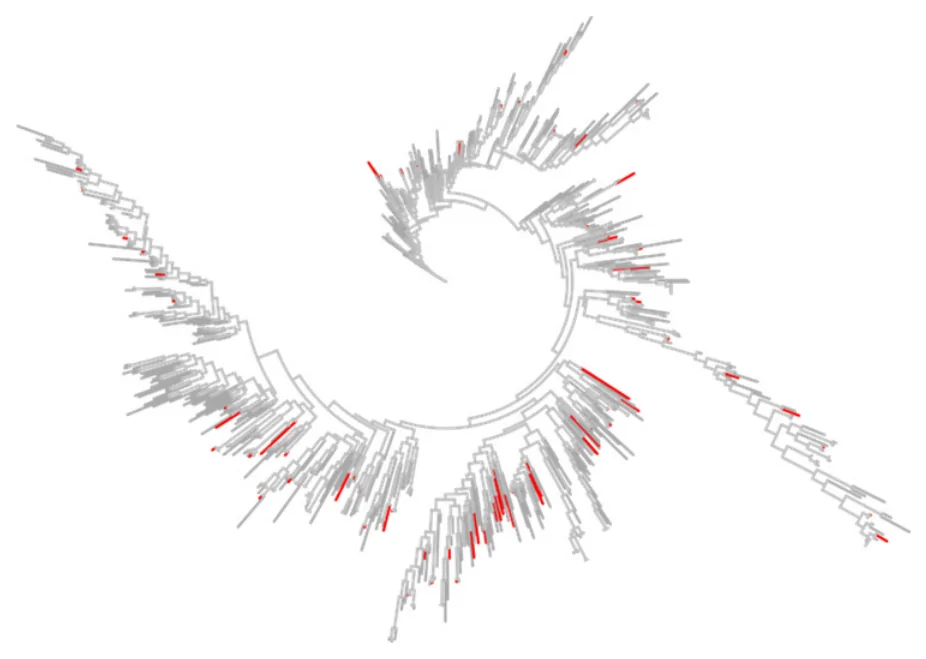
Distribution of the STPGA-selected training population of 100 individuals in the phylogenetic tree.

**Figure 8:**
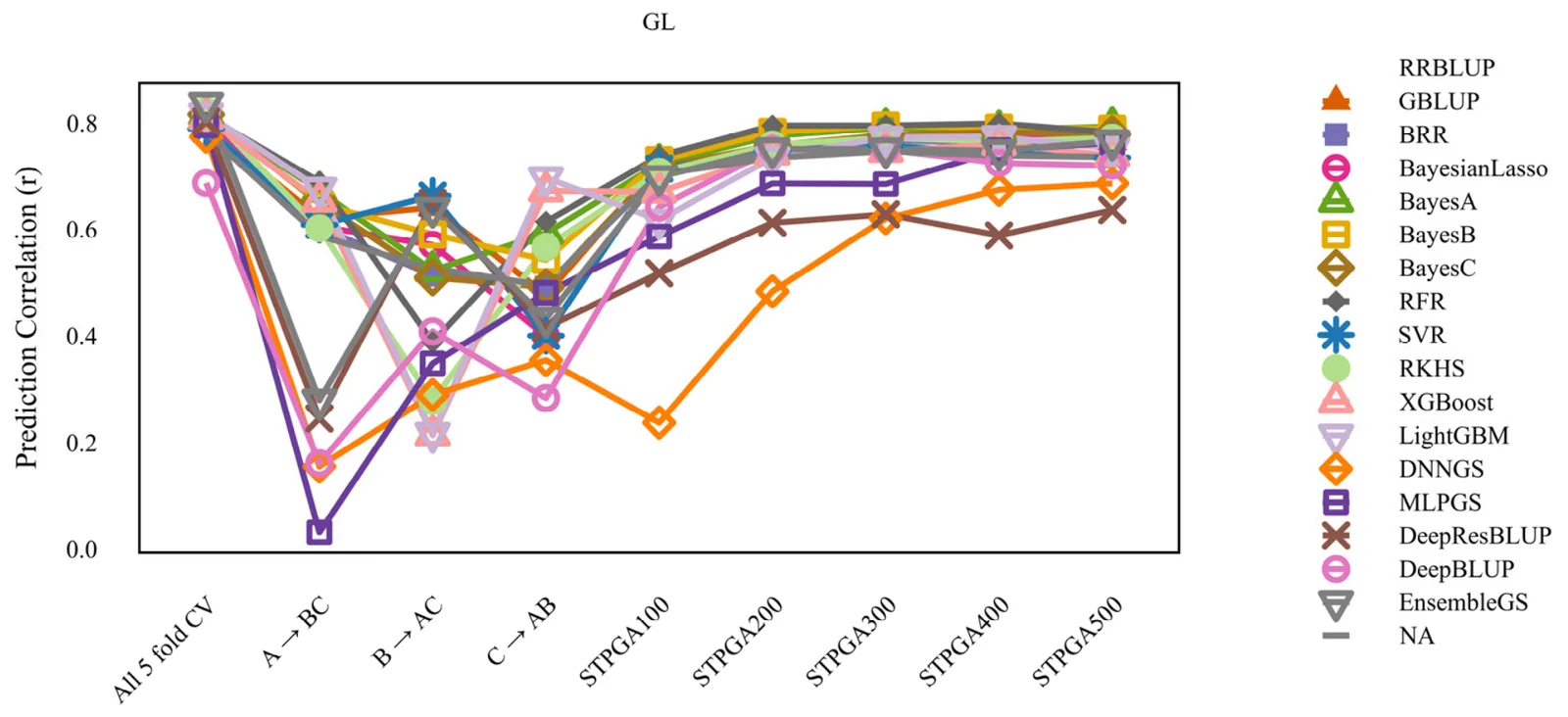
Comparison of predictive performance under different prediction strategies for grain length (GL). Full-population five-fold cross-validation, cross-population prediction using the A → BC, B → AC, and C → AB schemes, and STPGA-optimized training populations of different sizes were compared. In the cross-population prediction analysis, subgroups A, B, and C contained 314, 317, and 198 individuals, respectively. One subgroup was used as the training population and the remaining two subgroups were combined as the prediction target. Because subgroup sizes and target-population compositions differed among scenarios, the cross-population results were used to evaluate prediction transferability across genetically differentiated populations rather than to provide a strictly sample-size-controlled comparison. For STPGA, training populations of 100–500 individuals were selected, with the corresponding remaining accessions used as prediction targets. Results for the other seven traits are provided in Supplementary Figure S4.

**Figure 9:**
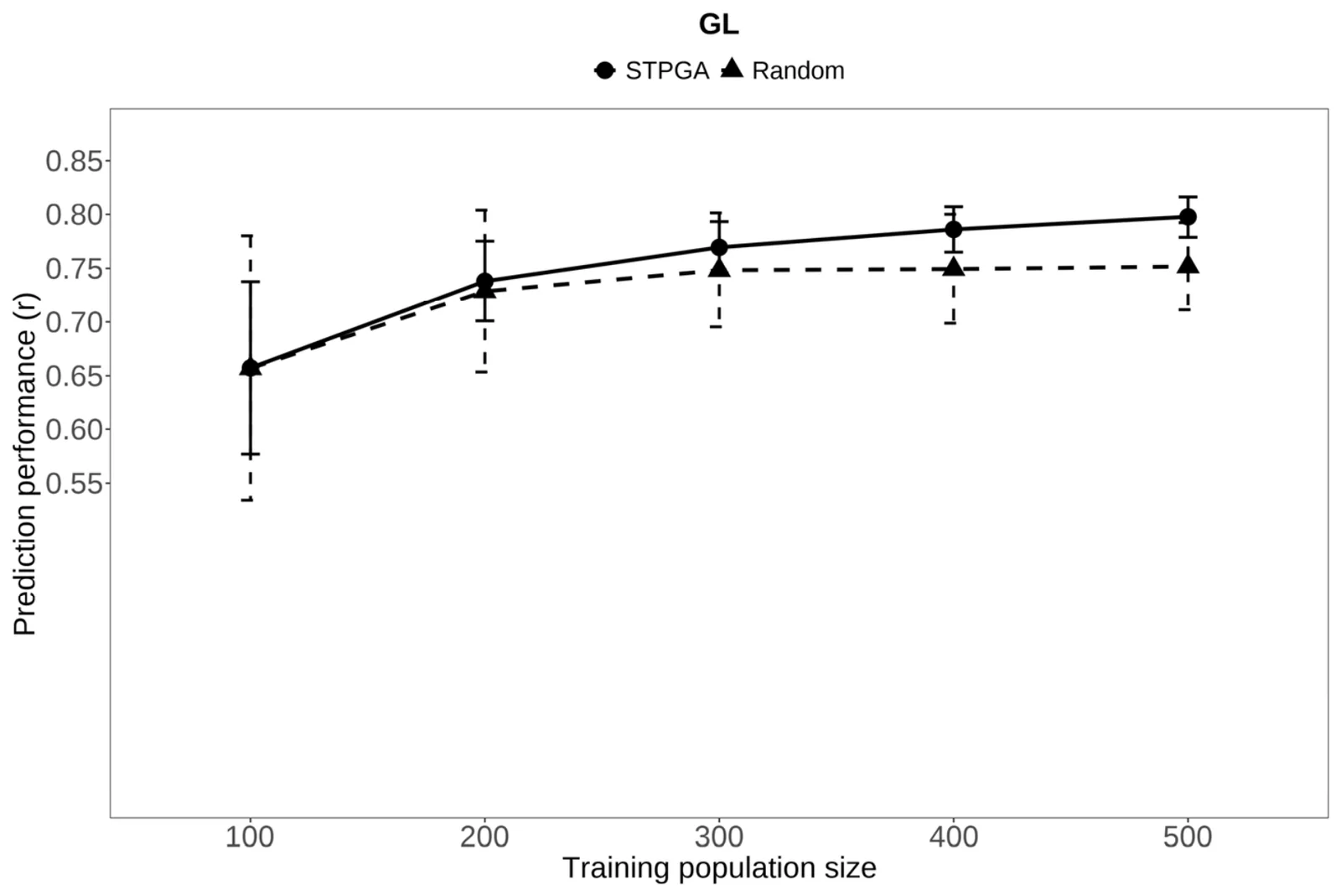
Comparison of prediction performance between STPGA-selected and randomly selected training populations for grain length (GL) across training population sizes of 100, 200, 300, 400, and 500 individuals. STPGA was independently repeated 10 times for each training population size, whereas random sampling was independently repeated 100 times. The plotted values represent the mean predictive performance across the 17 genomic prediction models, with prediction performance assessed using Pearson’s correlation coefficient (*r*). Error bars represent the standard deviation across the 17 model-specific predictive performance values. Results for the other seven traits are provided in Supplementary Figure S5.

**Figure 10:**
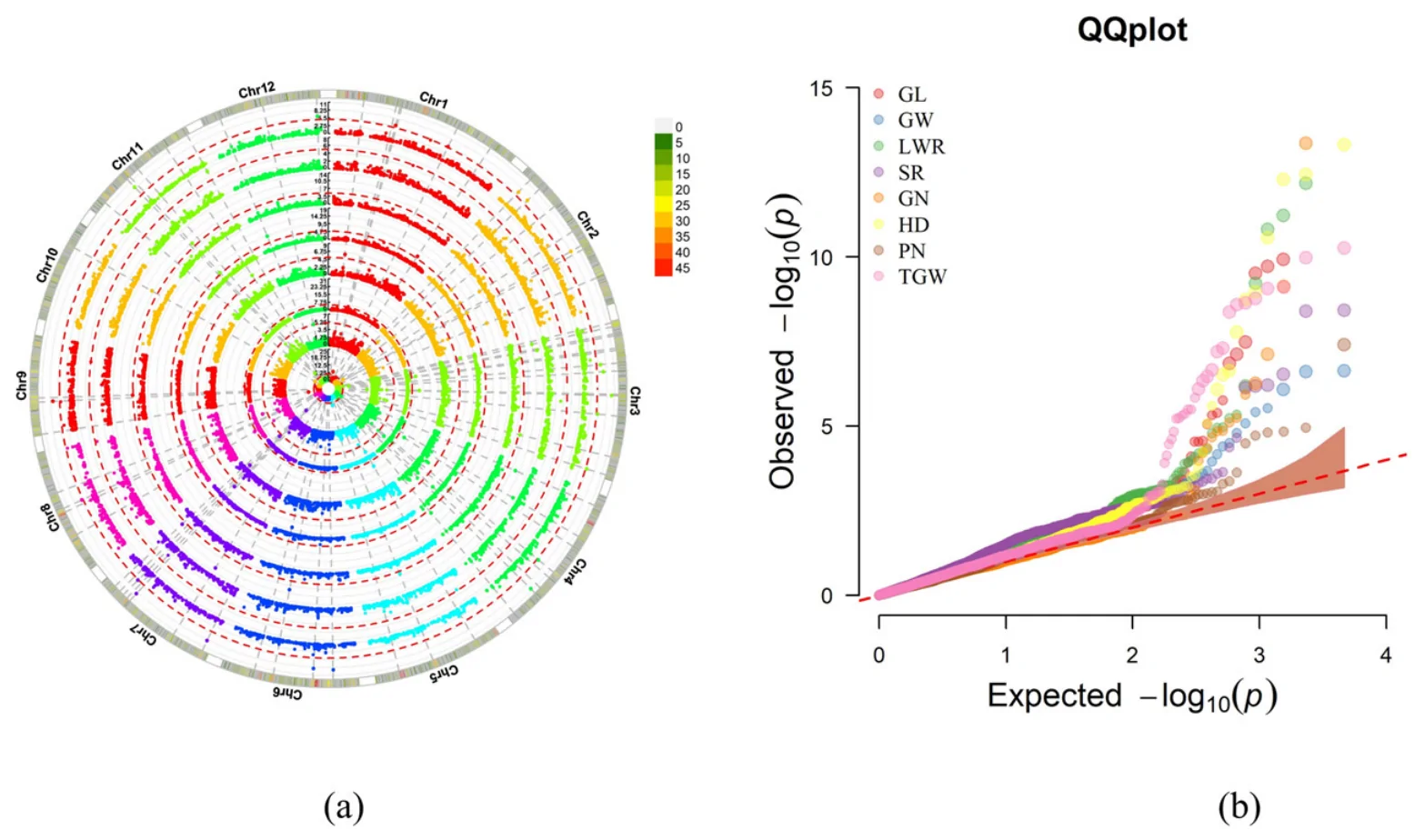
Genome-wide association analysis of the eight agronomic traits using the STPGA-optimized training population (n = 400). (**a**) Manhattan plots.one circle per trait (HD, GN, SR, GL, GW, LWR, TGW, PN); colors distinguish the 12 rice chromosomes. (**b**) Quantile–quantile (Q–Q) plots. The red dashed line is the null expectation (y = x); the orange shaded region is the 95% confidence envelope under the null model.

**Figure 11:**
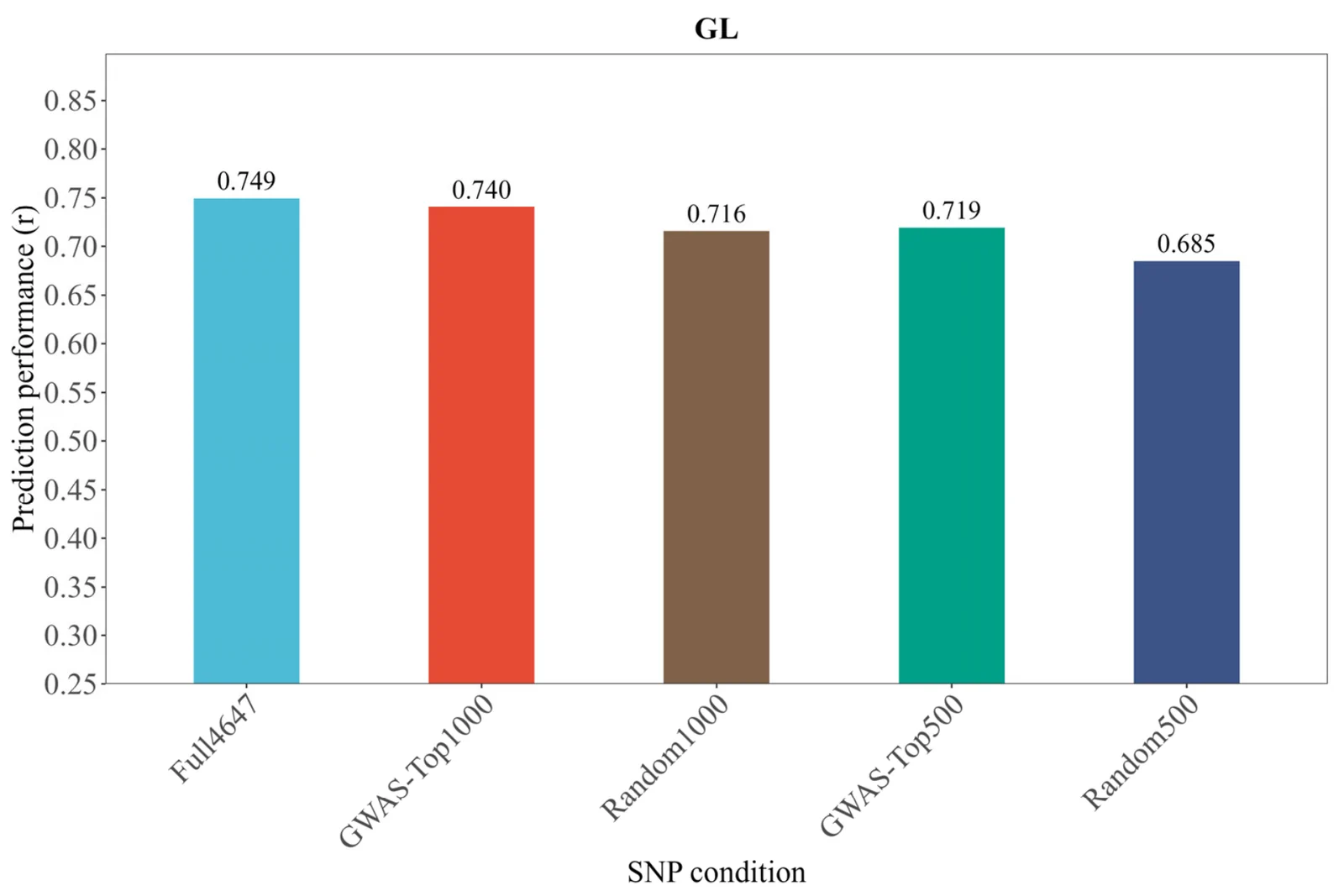
Predictive performance of genomic prediction using different SNP subsets under the STPGA-selected training population. A fixed training population of 400 individuals selected by STPGA was used to predict the remaining 429 individuals. Five marker conditions were evaluated: Full4647 (4647 SNPs), GWAS-Top1000, Random1000, GWAS-Top500, and Random500. Full4647, GWAS-Top1000, and GWAS-Top500 represent fixed SNP sets and therefore yield a single predictive-performance estimate for each analysis. Random1000 and Random500 represent 100 independently sampled random SNP subsets containing 1000 and 500 SNPs, respectively, and their predictive performance was summarized across the random replicates. Predictive performance was measured using Pearson’s correlation coefficient (*r*). Comparisons between GWAS-prioritized and randomly selected SNP subsets at the same marker density were used to evaluate the extent of predictive information retained under GWAS-based marker prioritization and random marker reduction. The Full4647 condition using the STPGA-selected 400-individual training population was included as the full-marker reference for evaluating predictive performance after marker reduction. Detailed numerical results are provided in Supplementary Table S6, and results for the other seven traits are provided in Supplementary Figure S6.

**Table 1:** Genomic selection models evaluated in this study.

Model	Category	Reference
RR-BLUP	Linear	[1]
GBLUP	Linear	[16]
BRR	Linear	[17]
BayesA	Bayesian	[1]
BayesB	Bayesian	[1]
BayesC	Bayesian	[18]
Bayesian Lasso	Bayesian	[19]
RKHS	Kernel	[20]
RFR	Machine learning	[21]
SVR	Machine learning	[22]
XGBoost	Machine learning	[23]
LightGBM	Machine learning	[24]
DNNGS	Deep learning	[15]
MLPGS	Deep learning	[15]
DeepResBLUP	Deep learning	[15]
DeepBLUP	Deep learning	[15]
EnsembleGS ^1^	Ensemble	[15]

^1^ An ensemble model (EnsembleGS) was constructed by integrating predictions from RR-BLUP, BRR, LightGBM, MLPGS, and DeepBLUP to improve overall predictive performance.

**Table 2:** Predefined trait-priority weights used for multi-trait SNP prioritization.

Trait	Weight
GN	2.0
TGW	2.0
PN	2.0
SR	1.5
GL	1.0
GW	1.0
LWR	0.5
HD	0.5

Note: Trait-priority weights were predefined before SNP ranking based on practical rice breeding experience and the breeding objectives considered in this study. The weights represent relative trait priorities for breeding-oriented SNP selection and were used solely for multi-trait SNP prioritization; they were not statistically estimated from the GWAS results and do not represent phenotypic measurement weights or quantitative effect sizes.

**Table 3:** Summary statistics of the eight agronomic traits.

Trait	Observed N	KNN-imputed N	Imputed (%)	Mean	SD	Range	CV (%)
GN (grains panicle^−1^)	828	1	0.12	160.34	42.91	52.00–397.00	26.76
TGW (g)	828	1	0.12	24.16	4.23	14.36–51.69	17.51
PN (panicles plant^−1^)	825	4	0.48	11.19	2.97	4.00–22.00	26.57
SR (proportion)	824	5	0.60	0.75	0.1	0.34–0.97	13.12
GL (mm)	829	0	0.00	9.2	0.83	6.84–13.54	9.05
GW (mm)	829	0	0.00	2.48	0.32	1.37–4.32	12.95
LWR (dimensionless)	829	0	0.00	3.86	0.59	2.00–5.90	15.26
HD (days)	822	7	0.84	88.26	8.17	55.00–111.00	9.26

Note: Missing phenotypic observations were imputed using a k-nearest neighbors (KNN) approach before downstream analyses. Observed N indicates the number of accessions with non-missing phenotypic observations in the original dataset, KNN-imputed N indicates the number of missing observations replaced by KNN-imputed values, and Imputed (%) represents the proportion of KNN-imputed observations relative to the total of 829 accessions. The descriptive statistics were calculated using the KNN-imputed dataset of 829 accessions.

## Data Availability

The raw data supporting the conclusions of this article will be made available by the authors on request.

## Supplementary Materials

The following supporting information can be downloaded at https://www.mdpi.com/article/10.3390/genes17101240/s1, Figure S1. Variation in predictive performance of 17 genomic selection models across eight agronomic traits under repeated five-fold cross-validation. Figure S2. Three-dimensional PCA distributions of STPGA-optimized training populations of 100, 200, 300, 400, and 500 individuals, showing the spatial separation between selected training sets and test sets along PC1, PC2, and PC3. Figure S3. Phylogenetic-tree distributions of STPGA-optimized training populations of 100, 200, 300, 400, and 500 individuals, showing the spread of selected individuals across genetic clusters. Figure S4. Comparison of predictive performance under different prediction strategies for the eight agronomic traits. Figure S5. Comparison of prediction performance between STPGA-selected and randomly selected training populations for the eight agronomic traits. Figure S6. Predictive ability using the full 4,647-SNP panel, GWAS-prioritized subsets (GWAS-Top500, GWAS-Top1000), and size-matched random subsets (Random500, Random1000) across the eight agronomic traits. Table S1. Prediction abilities (Pearson correlation coefficients, r) of genomic prediction models under five-fold cross-validation using the entire population. Table S2. Prediction abilities (Pearson correlation coefficients, r) of genomic prediction models under different cross-population prediction scenarios. Table S3. Prediction abilities (Pearson correlation coefficients, r) of genomic prediction models using STPGA-selected training populations of different sizes (100–500 individuals). Table S4. Prediction abilities (Pearson correlation coefficients, r) of genomic prediction models using randomly selected training populations of different sizes (100–500 individuals). Table S5. GWAS-derived P-values and integrated SNP scores used for marker selection in genomic prediction. Table S6. Predictive performance of 17 genomic selection models using GWAS-prioritized and randomly selected SNP subsets of different sizes. Table S7. Software implementations and key parameter settings of the genomic selection models evaluated in this study. Table S8. Parameters used for STPGA training population optimization.
